# Toe Grip strength declines earlier than hand grip strength and knee extension strength in community-dwelling older men: a cross sectional study

**DOI:** 10.1186/s13047-022-00584-x

**Published:** 2022-10-25

**Authors:** Sayo Miura, Toshiaki Seko, Nobuaki Himuro, Masayuki Koyama, Shigeyuki Saitoh, Hirofumi Ohnishi

**Affiliations:** 1 Department of Rehabilitation, Japan Health Care College, S-1, W-17, Chuo-ku, Sapporo, 060-8556 Japan; 2 Department of Public Health, Sapporo Medical University School of Medicine, 3-11-1-50, Tsukisamu Higashi, Toyohira-ku, Sapporo, Hokkaido 062-0053 Japan; 3 Department of Rehabilitation, Hokkaido Chitose College of Rehabilitation, 2-10 Satomi, Chitose, 066-0055 Japan; 4 Department of Cardiovascular, Renal and Metabolic Medicine, Sapporo Medical University School of Medicine, S-1, W-17, Chuo-ku, Sapporo, 060-8556 Japan

**Keywords:** Age group, Frail older adults, Geriatric assessment, Muscle strength, Toe grip

## Abstract

**Background:**

The aim of this study is to examine the age-related changes in the toe grip strength and its differences from hand grip strength and knee extension strength using cross-sectional data.

**Methods:**

Of participants aged 65 years over who underwent health checkups for lifestyle-related diseases in 2018, 307 men and women met the criteria. Toe grip strength, hand grip strength, and knee extension strength were also measured as optional tests. The participants were divided into five groups categorized by every 5 years of age (Group 65–85). The data were analyzed with multiple comparisons using the linear mixed multilevel model to examine the following categories: association between age and muscle strength, differences in the pattern of change, and gender, using the 65–69 years group as a reference.

**Results:**

In men, there were interaction effects between the factors of age and muscle, but in women there were not. Toe grip strength was significantly lower in Group 70, 75, 80, and 85 in men, lower in Group 85 than in 65 in women. Hand grip strength was significantly lower in Group 85 than in 65 in both men and women. There was no significant difference in knee extension strength among the age groups for both men and women.

**Conclusions:**

The decline in toe grip strength may occur earlier and in a different pattern from hand grip strength and knee extension strength in men.

**Supplementary Information:**

The online version contains supplementary material available at 10.1186/s13047-022-00584-x.

## Background

The toes and plantar surface of the feet of humans are the only the parts touching the ground while in a standing posture and they play very important roles in walking and posture control [1]. Toe grip strength is one of the important functions of the toes and plantar surface of the feet, which is the strength for gripping the ground by bending toes. Because toe grip strength is related to the balance ability and fall history of the older adults, it is important to maintain toe grip strength in the old age [2]. Since toe grip strength can be measured by using a simple dedicated device [3], it can be applied to screening tests at health checkups setting.

For prevention of frailty in older adults, it is important to identify high-risk individuals at an early stage according to indicators that sensitively reflect age-related weakness in muscle strength, as indicators of muscle strength in older adults, grip strength and knee extension strength have been commonly used until now [4–7]. In a previous study, toe grip strength significantly declines with aging after 50 s in men, it declines more slowly after 40-50 s in women than in men [8]. In addition, a study of healthy men in middle age reported that toe grip strength declined more significantly earlier than hand grip strength [9]. Therefore, toe grip strength may reflect early muscle weakness, which is at the core of sarcopenia and locomotive syndrome. Because locomotive syndrome is a comprehensive concept that includes muscular and nervous system dysfunction, joint deformation, bone weakness, gait disturbance, and need for nursing care. It is said that prevention of locomotive syndrome is useful for prevention of frailty, sarcopenia, and disorders secondary to them, and prevention and early detection of locomotive syndrome are also important for prevention of long-term care [10]. However, the difference in the timing of decline of toe grip strength compared with other muscle strength indices such as hand grip strength and knee extension strength in old age is still unknown.

The purpose of this study is to examine the age-related changes in toe grip strength, and the difference in age-related change compared with both hand grip strength and knee extension strength in community-dwelling older adults.

## Methods

### Participants

The Tanno-Sobetsu study is a prospective cohort study in which residents of two towns, Tanno and Sobetsu, have been recruited for annual health checkups for lifestyle-related diseases such as metabolic syndrome or type 2 diabetes since 1977. In the health checkups in Sobetsu town, the measurement of toe grip strength started in 2018 as an optional test. Of participants who received health checkups in 2018, 314 men and women, aged 65–94 years, received the test of toe grip strength. Participants with pain in the toe or knee joints during muscle strength measurement and those with severe deformity of the toe that made measurement difficult were excluded. Seven individuals were excluded because of incomplete data; thus, 307 participants were included in the current study. This study was approved by the ethics committee of Sapporo Medical University, and we received written informed consent from all participants.

### Measurements

Muscle strength was measured for toe grip strength (kg), hand grip strength (kg), and knee extension strength (kg). Toe grip strength was measured using a toe grip dynamometer (T.K.K. 3362, Takei Scientific Instruments, Niigata, Japan). The participants sat in a chair, with the trunk in the neutral position and the hip and knee joints in the 90 degrees flexion position throughout the measurement. The examiner adjusted the position of each participant's heel stopper so that at least the first to third toes could grasp the grip bar of the device, and secured the foot with the provided immobilization belt to prevent it from moving from that position. After practicing several times, toe grip strength of both sides was measured at the maximal force for about three seconds. The hand grip strength of both hands was measured using a Smedley type grip strength meter (Grip D; Takei Scientific Instruments, Niigata, Japan) while the participants were standing with the arms resting naturally. The examiner adjusted the device so that their second joint of the index finger was at 90 degrees, and ensured that the grip strength meter didn’t touch their body or clothes [11]. Knee extension strength of both sides was measured using a hand-held dynamometer (Mobie MT-100; SAKAI Med, Tokyo, Japan). The participants sat in a chair, with the trunk in the neutral position, the hip and knee joints in the 90 degrees flexion position, and the arms folded. The force sensor was fixed to the distal side of the lower leg by a belt. The examiner fixed the participant’s pelvis with both hands [12]. After practicing several times, knee extension strength of both sides was measured at the maximal force for about three seconds. Toe grip strength, hand grip strength, and knee extension strength were measured twice on both sides, and the average value (kg) was calculated from the maximum value on each side. All muscle strength measurements were performed by qualified physical therapists.

The serum biochemical parameters measured were Albumin, high-sensitivity C-reactive protein (hsCRP), estimated glomerular filtration (eGFR), total cholesterol (TC), low-density lipoprotein cholesterol (LDL-C), high-density cholesterol (HDL-C), and Hemoglobin A1c (HbA1c). The sociodemographic characteristics were collected for age, gender, height, weight, body mass index (BMI), systolic blood pressure (SBP), diastolic blood pressure (DBP), smoking habit (current/former/never) and alcohol drinking habit (usually/sometimes/never).

### Statistical analysis

Participants were divided into five groups according to their chronological age, 65–69 (Group 65), 70–74 (Group 70), 75–79 (Group 75), 80–84 (Group 80), ≥ 85 (Group 85) years. Multiple comparisons were used to investigate whether there were significant differences in physical characteristics, serum biochemical parameters, smoking and drinking habits, and muscle strength variables for each age group with Group 65 as the reference separately for men and women.

The linear mixed modeling approach was used to examine the association between age and muscle strength with the residual maximum likelihood estimation method. The interaction effects were used to examine whether differences in the patterns of change were present between each muscle strength group with aging. The confounding factors were selected to minimize Akaike's information criterion (AIC) and the models were run separately for men and women. In both men and women, age groups were included as fixed effects, and the participant was included as a random effect. As confounding factors, several serum biochemical parameters were used in the analysis. Sarcopenia defined as a decrease in muscle mass and strength due to aging is known to be caused by a combination of various factors, such as oxidative stress, aging, inactivity, altered metabolic demand, insulin resistance, decreased dietary intake, changes in hormones, and others [13]. Moreover, the health status of older adults, in particular, varies among individuals. Due to organ status and medical history, the general condition varies from individual. Therefore, in order to remove the influence of age-related changes in an individual’s general condition, such serum biochemical parameters were included as covariates. As a result of examining optimal model construction by AIC, in men, BMI of the obesity index and hsCRP of the inflammation index were selected as confounding factors. In women, BMI, SBP and DBP of cardiovascular disease index, albumin of the nutrition index, hsCRP of the inflammation index, eGFR of renal function index, LDL-C and HDL-C of the lipid index, and HbA1c of blood glucose index were selected as confounding factors to eliminate effects of the aging in individuals due to aging change. The muscle strength values preliminarily confirmed that there was a high correlation between the left and right muscle strength values in each muscle group. (*r* = 0.82–0.90) After confirming this, the average value of the right and left maximum values was adopted for analysis. Furthermore, multiple comparisons by Bonferroni method was used. IBM SPSS statistics version 24 (Armonk, NY, USA) and EZR (Saitama Medical center, Jichi Medical University, Saitama, Japan) which is a graphical user interface for R (The R Foundation for Statistical Computing, Vienna, Austria) were used for statistical analysis. The significance level in all analysis was set at *P* < 0.05.

## Results

Tables 1 and 2 shows the characteristics of this study population for men and women. In men, the mean and median BMI, SBP, DBP, Albumin, hsCRP, eGFR, TC, LDL-C, HDL-C, HbA1c, smoking habit, and alcohol drinking habit were no significant differences among the age groups when Group 65 was used as a reference. The mean toe grip strength was significantly different from Group 65 in Group 75, 80, and 85, hand grip strength was significantly different between Group 80 and 85, and knee extension strength was significantly different only for Group 85. In women, all biochemical data except TC, smoking habit, and alcohol drinking habit were not significantly different among the age groups; TC was lower in Group 85. The mean toe grip strength and hand grip strength were significantly different from Group 65 in Group 80 and 85, but knee extension strength had no significant difference.

**Table 1:** Characteristics of study population for men

	**Group 65 (65–69) (*n* = 34) Ref**	**Group 70 (70–74) (*n* = 37)**	**Group 75 (75–79) (*n* = 32)**	**Group 80 (80–84) (*n* = 28)**	**Group 85 (≧85) (*n* = 10)**	***P-value***
**Age (years)**	67.0 (66.0–68.0)	71.0 (70.5–73.0)^a^	77.0 (76.0–78.0) ^a^	82.0 (80.0–83.0) ^a^	88.0 (85.8–89.0) ^a^	< 0.001
**Height (cm)**	165.1 ± 5.0	165.1 ± 4.8	160.1 ± 5.7 ^a^	159.3 ± 6.3 ^a^	159.5 ± 7.9 ^a^	< 0.001
**Body Weight (kg)**	63.7 (57.9–72.7)	64.5 (59.8–74.5)	62.9 (56.2–73.4)	59.1 (47.5–62.6) ^a^	54.4 (43.3–68.1)	0.002
**BMI**	23.4 (20.7–26.4)	23.9 (22.1–26.6)	23.9 (22.1–27.5)	21.9 (19.9–24.2)	21.5 (18.8–25.4)	0.027
**SBP (mmHg)**	133.1 ± 23.5	133.1 ± 20.8	129.9 ± 38.1	141.2 ± 20.5	131.4 ± 18.0	0.381
**DBP (mmHg)**	72.2 ± 12.7	75.5 ± 9.9	72.2 ± 12.1	77.7 ± 10.6	75.0 ± 5.6	0.263
**Biochemical data**						
Albumin (g/dL)	4.3 (4.2–4.5)	4.4 (4.3–4.5)	4.4 (4.3–4.5)	4.4 (4.2–4.6)	4.5 (4.2–4.7)	0.639
hsCRP (mg/dL)	0.03 (0.02–0.08)	0.07 (0.03–0.18)	0.04 (0.02–0.10)	0.04 (0.02–0.07)	0.03 (0.01–0.07)	0.390
eGFR (mL/min/1.73m^2^)	64.6 (55.9–74.0)	65.1 (56.8–75.2)	66.5 (55.7–73.8)	65.7 (57.9–74.9)	62.9 (55.5–75.5)	0.980
TC (mg/dL)	203.0 (174.5–227.5)	195.0 (178.5–225.5)	195.0 (175.0–234.0)	200.0 (184.3–222.8)	181.0 (172.8–204.5)	0.689
LDL-C (mg/dL)	111.0 (96.0–137.5)	112.0 (96.0–143.0)	112.0 (97.0–149.0)	115.5 (102.8–136.8)	95.0 (86.3–106.8)	0.200
HDL-C (mg/dL)	67.0 (58.0–77.5)	59.0 (50.5–70.0)	69.0 (56.0–84.0)	60.5 (56.0–77.0)	67.0 (48.5–85.3)	0.084
HbA1c (%)	5.6 (5.3–5.9)	5.5 (5.3–5.9)	5.7 (5.5–5.8)	5.6 (5.4–5.9)	5.4 (5.3–5.8)	0.619
**Smoking habit**						
Current/former/never (%)	23.5/55.9/17.6	24.3/59.5/16.2	12.9/67.7/19.4	14.3/67.9/17.9	50.0/40.0/10.0	0.293
**Alcohol drinking habit**						
Usually/sometimes/never (%)	29.4/14.7/52.9	16.2/27.0/56.8	9.7/29.0/61.3	21.4/21.4/57.1	20.0/20.0/60.0	0.870
**Muscle strength (kg)**						
Toe grip strength	18.75 ± 4.90	15.36 ± 6.01	12.94 ± 4.23 ^a^	11.64 ± 5.53 ^a^	8.12 ± 3.85 ^a^	< 0.001
Hand grip strength	40.25 ± 8.08	38.88 ± 6.16	37.32 ± 7.71	32.57 ± 7.60 ^a^	26.28 ± 6.84 ^a^	< 0.001
Knee extension strength	30.38 ± 9.56	31.34 ± 8.61	30.39 ± 9.02	25.49 ± 9.15	19.69 ± 10.06 ^a^	0.003

Data are presented as the median (interquartile ranges) or mean ± standard deviation

*BMI* Body mass index, *SBP* Systolic blood pressure, *DBP* Diastolic blood pressure, *hsCRP* High-sensitivity C-reactive protein, *eGFR* Estimated glomerular filtration rate, *TC* Total cholesterol, *LDL-C* Low-density lipoprotein cholesterol, *HDL-C* High-density lipoprotein cholesterol, *HbA1c* Hemoglobin A1c

*P* values from One-way analysis of variance or Kruskal–Wallis test

^a^significant difference from Group 65 as a reference

**Table 2:** Characteristics of study population for women

	**Group 65 (65–69) (*n* = 41) Ref**	**Group 70 (70–74) (*n* = 42)**	**Group 75 (75–79) (*n* = 39)**	**Group 80 (80–84) (*n* = 29)**	**Group 85 (≧85) (*n* = 15)**	***P-value***
**Age (years)**	67.0 (66.0–69.0)	72.0 (70.0–74.0) ^a^	77.0 (75.0–78.0)^a^	81.0 (81.0–83.0) ^a^	88.0 (85.0–89.0) ^a^	< 0.001
**Height (cm)**	151.9 ± 5.8	149.3 ± 5.8	148.7 ± 4.6	147.1 ± 5.9 ^a^	143.7 ± 4.5 ^a^	< 0.001
**Body Weight (kg)**	51.9 (45.3–59.1)	52.8 (44.0–56.2)	50.0 (46.5–58.8)	48.9 (43.1–55.4)	45.4 (40.4–48.4) ^a^	0.030
**BMI**	23.0 (20.7–25.5)	22.8 (21.3–25.8)	23.1 (20.6–26.4)	22.4 (20.1–25.2)	21.5 (20.9–23.6)	0.573
**SBP (mmHg)**	130.7 ± 20.3	132.4 ± 21.8	133.9 ± 18.5	137.1 ± 18.4	136.3 ± 21.1	0.713
**DBP (mmHg)**	72.8 ± 12.0	75.0 ± 10.6	70.7 ± 10.7	76.8 ± 11.7	73.1 ± 12.7	0.239
**Biochemical data**						
Albumin (g/dL)	4.4 (4.3–4.6)	4.5 (4.3–4.5)	4.4 (4.2–4.5)	4.3 (4.2–4.5)	4.3 (4.1–4.4)	0.022
hsCRP (mg/dL)	0.03 (0.01–0.06)	0.04 (0.28–0.75)	0.05 (0.03–0.08)	0.05 (0.02–0.07)	0.04 (0.02–0.09)	0.453
eGFR (mL/min/1.73m^2^)	65.0 (60.9–70.4)	64.6 (59.1–70.5)	59.3 (52.1–67.5)	62.3 (53.8–68.1)	63.8 (55.4–70.9)	0.069
TC (mg/dL)	200.0 (176.0–212.5)	202.5 (183.0–228.3)	183.5 (150.5–212.0)	189.0 (170–209.5)	162.0 (158.0–193.0) ^a^	0.003
LDL-C (mg/dL)	114.0 (92.5–134.5)	119.5 (100.8–135.0)	104.5 (87.3–132.8)	107.0 (92.5–129.0)	91.0 (81.0–119.0)	0.036
HDL-C (mg/dL)	62.0 (50.0–71.0)	63.5 (54.0–79.0)	51.5 (42.3–69.0)	60.0 (54.5–64.5)	63.0 (48.0–65.0)	0.039
HbA1c (%)	5.5 (5.3–5.7)	5.6 (5.4–5.8)	5.5 (5.4–6.1)	5.6 (5.4–5.8)	5.7 (5.3–5.9)	0.309
**Smoking habit**						
Current/former/never (%)	14.6/53.7/31.7	4.8/71.4/23.8	10.0/62.5/27.5	6.9/62.1/31.0	13.3/66.7/20.0	0.929
**Alcohol drinking habit**						
Usually/sometimes/never (%)	19.5/29.3/48.8	26.2/33.3/40.5	17.5/22.5/60.0	17.2/24.1/58.6	20.0/6.7/73.3	0.284
**Muscle strength (kg)**						
Toe grip strength	12.19 ± 4.03	10.97 ± 4.01	10.45 ± 3.49	9.20 ± 3.41 ^a^	6.93 ± 3.14 ^a^	< 0.001
Hand grip strength	25.85 ± 4.80	23.51 ± 5.15	23.62 ± 4.58	22.67 ± 3.22 ^a^	19.23 ± 3.42 ^a^	< 0.001
Knee extension strength	21.54 ± 6.37	19.37 ± 6.42	19.77 ± 8.63	18.46 ± 4.92	16.55 ± 4.20	0.170

Data are presented as the median (interquartile ranges) or mean ± standard deviation

*BMI* Body mass index, *SBP* Systolic blood pressure, *DBP* Diastolic blood pressure, *hsCRP* High-sensitivity C-reactive protein, *eGFR* Estimated glomerular filtration rate, *TC* Total cholesterol, *LDL-C* Low-density lipoprotein cholesterol, *HDL-C* High-density lipoprotein cholesterol, *HbA1c* Hemoglobin A1c

*P* values from One-way analysis of variance or Kruskal–Wallis test

^a^significant difference from Group 65 as a reference

**Fig. 1:**
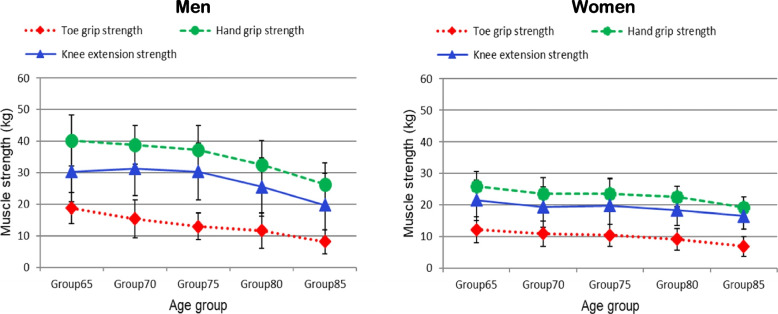
The change of the mean muscle strength of toe grip strength, hand grip strength, and knee extension strength by age group

Figure 1 shows the changes in muscle strength from Group 65 to Group 85. As the age category increased, each index of muscle strength showed a decreasing association.

Table 3 shows the results of linear mixed-effects model analysis after adjustment for selected confounding factors based on AIC (Additional files 1 and 2). For men, Model 2 was adopted based on AIC, while Model 4 was adopted for women. There was a significant interaction between age categories and indices of muscle strength in men (*P* = 0.049) but not in women (*P* = 0.989).

**Table 3:** Linear mixed effect model between age group and muscle strength and the interaction effects

	**Men**					**Women**				
	**Estimate**	**95%CI**	**SE**	**t**	***P-value***	**Estimate**	**95%CI**	**SE**	**t**	***P-value***
**(Intercept)**	10.026	1.448 to 18.605	4.338	2.311	0.022	27.482	8.397 to 46.567	9.634	2.853	0.005
**Age group**	-2.0899	-2.877 to -1.303	0.396	-5.281	< 0.001	-1.182	-1.758 to -0.629	0.285	-4.188	< 0.001
**Muscle strength group**	11.428	10.595 to 12.260	0.420	27.198	< 0.001	6.966	6.511 to 7.426	0.231	30.097	< 0.001
**Age group × Muscle strength group**	-0.202	-3.082 to -0.454	0.326	-0.619	0.049	-0.131	-0.504 to 0.242	0.189	-0.692	0.989

Model were adjusted by body mass index, high-sensitivity C-reactive protein in men

Model were adjusted by body mass index, systolic blood pressure, diastolic blood pressure, Albumin, high-sensitivity C-reactive protein, estimated glomerular filtration rate, low-density lipoprotein cholesterol, high-density lipoprotein cholesterol, Hemoglobin A1c, smoking habit, alcohol drinking habit in women

*P* values from linear mixed effect model

Table 4 shows the results of multiple comparisons of muscle strength adjusted by confounding factors. In men, toe grip strength was significantly lower in Group 70, 75, 80, and 85 than in Group 65. Hand grip strength was significantly lower in Group 85 than in Group 65. There was no significant difference in knee extension strength among the factors of age groups. In women, toe grip strength was significantly lower in Group 85 than in Group 65. Hand grip strength was significantly lower in Group 85 than in Group 65. There was no significant difference in knee extension strength among the age categories.

**Table 4:** Mean and standard deviation for the each muscle strength between age groups by gender using linear mixed multilevel models

**Muscle strength group (kg)**	**Men**					
	**Group 65 (*n* = 34)**	**Group 70 (*n* = 37)**	**Group 75 (*n* = 32)**	**Group 80 (*n* = 28)**	**Group 85 (*n* = 10)**	***P-value***
**Toe grip strength**	18.75 ± 4.90	15.36 ± 6.01 ^a^	12.94 ± 4.23^a^	11.64 ± 5.53 ^a^	8.12 ± 3.85 ^a^	< 0.001
**Hand grip strength**	40.25 ± 8.08	38.88 ± 6.16	37.32 ± 7.71	32.57 ± 7.60	26.28 ± 6.84 ^a^	
**Knee extension strength**	30.38 ± 9.56	31.34 ± 8.61	30.39 ± 9.02	25.49 ± 9.15	19.69 ± 10.06	
**Muscle strength group (kg)**	**Women**					
	**Group 65 (*n* = 41)**	**Group 70 (*n* = 42)**	**Group 75 (*n* = 39)**	**Group 80 (*n* = 29)**	**Group 85 (*n* = 15)**	***P-value***
**Toe grip strength**	12.19 ± 4.03	10.97 ± 4.01	10.45 ± 3.49	9.20 ± 3.41	6.93 ± 3.14 ^a^	0.002
**Hand grip strength**	25.85 ± 4.80	23.51 ± 5.15	23.62 ± 4.58	22.67 ± 3.22	19.23 ± 3.42 ^a^	
**Knee extension strength**	21.54 ± 6.37	19.37 ± 6.42	19.77 ± 8.63	18.46 ± 4.92	16.55 ± 4.20	

Data are presented as the mean ± standard deviation

Adjusted by body mass index, high-sensitivity C-reactive protein in men

Adjusted by body mass index, systolic blood pressure, diastolic blood pressure, Albumin, high-sensitivity C-reactive protein, estimated glomerular filtration rate, low-density lipoprotein cholesterol, high-density lipoprotein cholesterol, Hemoglobin A1c, smoking habit, alcohol drinking habit in women

*P* values from One-way analysis of variance or Kruskal–Wallis test

^a^significant difference from Group 65 as a reference

## Discussion

The decline in toe grip strength may occur earlier and show a different pattern from hand grip strength and knee extension strength in men.

The results of the present study were consistent in that the decline in toe grip strength occurs earlier than in hand grip strength with a previous study examining age-related changes in the toe grip strength and hand grip strength in middle-aged men [9]. The causes of age-related muscle weakness in older adults have reported pathophysiological factors such as neuronal denervation and loss, decreased anabolic, increased catabolic, decreased rates of muscle protein synthesis, and decreased hormone levels [14]. There was a tendency for muscle strength values to decrease with increasing age in both men and women for all toe grip strength, hand grip strength, and knee extension strength values in this study. Therefore, it is possible that the three muscles were commonly affected by these age-related pathophysiological factors. However, muscle strength declines differ depending on the muscle region and muscle, and skeletal muscle strength is more susceptible to age-related muscle weakness in the lower limbs than upper limbs [15, 16]. Even if lower limb muscle weakness occurs, the upper limb muscles are used more frequently because relying on the upper limb for movements is relatively easy to maintain. While the effects of aging are common to all muscle groups, they are also affected by the specific parts and sizes of each muscle, so there was a difference in the age groups that showed a decline.

In a previous study, there were different predictors of decline between toe grip strength and hand grip strength, specifically, metabolic disorders, which were independent predictors of toe grip strength, but not hand grip strength [9]. This might mean that age-related change affects muscle strength differently in each muscle, and there have also been different patterns between the two muscles in this study. Although hand grip strength is a predictor of various diseases or mortality has been used in many epidemiological studies [17–20], this study suggested toe grip strength is more suitable as an indicator to detect muscle weakness at an earlier stage.

The results from this cross-sectional study also suggested that a decline in the toe grip strength, in addition to that of hand grip strength, may appear from an earlier age in older adults than that in knee extension strength. Age-related changes in muscle strength tend to progress from distal to proximal [21]. Therefore, it is possible that the distal toe grip strength changed earlier than the knee extension strength. Toe grip strength and knee extension strength are both lower limb muscles, but there are no reports comparing the effects of aging. Knee extension strength declines at an accelerated rate in the 60 s, and at 85 years or older, it declines to less than 50% of its level in the 20 s [22]. Knee extension strength is not only involved in posture maintenance as an antigravity muscle, but also affects walking speed, which is an index of walking ability, and is associated with reduced mobility [23, 24]. Because of their role in supporting the lower limbs, the muscles are used more frequently on a daily basis. On the other hand, many older people have some foot problems, with a high rate of symptoms in the foot, toe deformities, skin lesions and nail problems [25–27]. It has been confirmed by ultrasound imaging that the thickness of intrinsic muscle is decreased in older adults with hallux valgus and lesser toe deformity [28, 29], and these deformities affect the balance, motor functions and the reaction time of flexor muscle groups of the toes [30]. In this study, we excluded the participants with deformities severe enough to make measurement difficult, but we cannot deny the possibility that we included those who had slight deformities but were still able to perform the measurement. However, the results may reflect the characteristics of the majority of older adults.

The specific age of decline in toe grip strength in this study did not match the specific age of decline in previous studies that examined age-related changes in toe grip strength in men and women aged 20–79 years [8]. In the present study, in order to examine the differences in the timing of decline of each muscle strength index in old age, the subjects were ≥ 65 years and the methods of statistical analysis are also different from the previous study. Therefore, the lack of consistent findings could be explained by several study-differences, such as differences in participants that are not entirely comparable.

In this study, the age at which toe grip strength significantly declined differed between men and women. In men, it was earlier than in women. One possible reason is that the secretion of testosterone in men declines with age and affects muscle strength [31, 32], and muscle strength in men changed to a much lower level than that in women. Furthermore, in this study, as the characteristics of toe grip strength by gender, because the previous value in men was larger than that in women, the extent of change in men becomes larger. It is also necessary to investigate whether there are differences in the age-related changes in toe grip strength between men and women by a longitudinal study.

There were several limitations to the present study. First, as this was a cross-sectional study, the translation of muscle strength decline of individuals was not clarified. It is undeniable that the results may have depended on the characteristics of the participants in this study. Furthermore, in this study, it was challenging to adopt a rigorous protocol in measuring of muscle strength because muscle strength was measured in health checkups as optional tests. Secondly, the effects of toe grip strength on geriatric syndrome and disease such as sarcopenia and frailty had not been investigated. A previous report had showed that among skeletal muscles, lower limb muscle strength had a greater relation to insulin resistance compared to upper limb, but in this study, we did not examine the relationship between toe grip strength and sarcopenia or diabetes mellitus [33]. In the future, the relationship between disease and toe grip strength, including the contribution of toe grip strength and cutoff values to skeletal muscle dysfunction, needs to be clarified through longitudinal studies. Finally, the participants were coming to the site of health checkups by walking, car and public transportation, and many of participants were engaged in agriculture, or other physically active work, so they were comparatively healthy. Therefore, it is possible that it included the self-selection bias.

## Conclusions

Toe grip strength in men possibly declines earlier than that of women in old age. Furthermore, toe grip strength in men tends to decline earlier and more remarkably than hand grip strength and knee extension strength. Further longitudinal studies are needed to clarify age-related changes in the toe grip strength.

## Supplementary Information

**Additional file 1. **Model comparison of linear mixed effect model in men.

**Additional file 2. **Model comparison of linear mixed effect model in women.

## Data Availability

The datasets used and/or analyzed during the current study are available from the corresponding author on reasonable request.

## Abbreviations

hsCRP: High-sensitivity C-reactive protein
eGFR: Estimated glomerular filtration
TC: Total cholesterol
LDL-C: Low-density lipoprotein cholesterol
HDL-C: High-density cholesterol
HbA1c: Hemoglobin A1c
BMI: Body mass index
SBP: Systolic blood pressure
DBP: Diastolic blood pressure
AIC: Akaike's information criterion
