# Correction: Toe grip strength declines earlier than hand grip strength and knee extension strength in community‑dwelling older men: a cross sectional study

**DOI:** 10.1186/s13047-022-00587-8

**Published:** 2022-11-08

**Authors:** Sayo Miura, Toshiaki Seko, Nobuaki Himuro, Masayuki Koyama, Shigeyuki Saitoh, Hirofumi Ohnishi

**Affiliations:** 1 Department of Rehabilitation, Japan Health Care College, 3-11-1-50, Tsukisamu Higashi, Toyohira-ku, 062-0053 Sapporo, Hokkaido Japan; 2 Department of Public Health, Sapporo Medical University School of Medicine, S-1, W-17, Chuo-ku, 060-8556 Sapporo, Japan; 3 Department of Rehabilitation, Hokkaido Chitose College of Rehabilitation, 2‑10 Satomi, 066‑0055 Chitose, Japan; 4 Department of Cardiovascular, Renal and Metabolic Medicine, Sapporo Medical University School of Medicine, S‑1, W‑17, Chuo‑ku, 060‑8556 Sapporo, Japan

**Correction: J Foot Ankle Res 15, 79 (2022)**

**https://doi.org/10.1186/s13047-022-00584-x**

Following publication of the original article [1], author found two minor errors in Affiliation 1 and Affiliation 2 addresses. The correct addresses are as follows:

- Aff1 "3-11-1-50, Tsukisamu Higashi, Toyohira-ku, Sapporo, Hokkaido, 062-0053, Japan"
- Aff2 "S-1, W-17, Chuo-ku, Sapporo, 060-8556, Japan"

The correction does not have any effect on the results or conclusions of the paper. The original article has been corrected.
